# Quality of Maternal Parenting of 9-Month-Old Infants Predicts Executive Function Performance at 2 and 3 Years of Age

**DOI:** 10.3389/fpsyg.2017.02293

**Published:** 2018-01-09

**Authors:** Nanhua Cheng, Shan Lu, Marc Archer, Zhengyan Wang

**Affiliations:** ^1^School of Psychology, Capital Normal University, Beijing, China; ^2^Institute of Psychology, Chinese Academy of Sciences, Beijing, China; ^3^Department of Psychology, University of the Chinese Academy of Sciences, Beijing, China; ^4^Center for Child Development, Capital Normal University, Beijing, China; ^5^Beijing Key Laboratory of Learning and Cognition, Capital Normal University, Beijing, China; ^6^School of Public Health, Zhejiang University, Hangzhou, China

**Keywords:** maternal parenting, maternal mind-mindedness, encouragement of autonomy, maternal sensitivity, executive function

## Abstract

Whereas the effects of maternal parenting quality during infants’ 2nd year on later executive function (EF) have been studied extensively, less is known about the impact of maternal parenting quality during the 1st year. The aim of this study was to examine whether maternal parenting during infants’ 1st year predicted EF performance at 2 and 3 years of age in a Chinese sample. Data were collected from 96 mother-infant dyads (42 males) when the infants were 6, 9, 25, and 38 months old. Cognitive development as a control variable was measured with the Bayley Scales of Infant Development II at 6 months. At 9 months, three aspects of maternal parenting quality (sensitivity, mind-mindedness, and encouragement of autonomy) were assessed with MBQS, mind-mindedness coding system, and encouragement of autonomy coding schema within a 15-min mother–infant interaction. Three aspects of EF (working memory, inhibitory control, and delay EF) were measured at 25 and 38 months with age-appropriate tasks. Hierarchical regression analysis showed that maternal mind-mindedness had a more important effect than did the encouragement of autonomy and maternal sensitivity during infants’ preverbal period. More precisely, maternal mind-mindedness at 9 months predicted inhibitory control at 2 and 3 years, and maternal encouragement of autonomy predicted performance on delay EF tasks at 3 years, maternal sensitivity had no observed effect on children’s EF. This study suggests that maternal parenting quality during the 1st year (maternal mind-mindedness and encouragement of autonomy, but not maternal sensitivity) impacts later EF development.

## Introduction

Executive function (EF) has been defined as a range of higher-order cognitive functions serving to control consciousness and behavior (Carlson, 2005; Garon et al., 2008). It includes the ability to set goals and accomplish these goals by coordinating thought and action with three component systems: working memory, inhibitory control, and cognitive flexibility (Garon et al., 2008; Hendry et al., 2016). EF in early childhood has been demonstrated to be responsible for various aspects of later child development, including language skills (Kuhn et al., 2014), academic performance (Bull and Lee, 2014), theory of mind (Benson et al., 2013), and behavioral problems (Sulik et al., 2015). The neurological underpinnings of EF include maturation of the prefrontal cortex, which occurs gradually from infancy to adolescence (Diamond, 2013). In addition to ongoing brain development, the effects of social interaction and the wider environment on EF formation are increasingly being studied (Moriguchi, 2014).

The influences of parent-infant interaction experiences, such as the early care environment (Miller and Marcovitch, 2015) and the quality of mother–infant interaction (Roskam et al., 2014), also provide a new perspective for training and intervention when delays in EF are identified. Considerable evidence suggests that EF has social origins and emerges in the context of social interaction (Moriguchi, 2014), particularly mother–infant interaction (Carlson, 2009). Several studies have identified positive correlations between the development of EF and maternal parenting, concurrently and longitudinally in different age ranges (e.g., 12–15-month-old infants: Bernier et al., 2010; Bernier et al., 2012; 2- and 3-year-old toddlers: Hughes and Ensor, 2009; Hammond et al., 2012; 4-year-old preschoolers: Landry et al., 2002). In addition, longitudinal positive associations have been found between maternal sensitivity when children are 3 years old and parent-rated EF performance at 4 years of age (Kok et al., 2014). However, the influence of maternal parenting in infants aged < 1 year has remained unexamined or underreported. Although Sulik et al. (2015) reported on longitudinal relationships between parenting of children aged 6–36 months and executive performance on EF tasks in children aged 36–60 months, they averaged the parenting period examined into a composite index, which limits the ability to examine the unique effects of parenting before the age of 1 year. Thus, the current study expands on previous research by examining the effects of maternal parenting quality during this preverbal stage on later EF at 2 and 3 years of age.

A wide range of maternal parenting behaviors has different effects on child EF outcomes (Bernier et al., 2010; Fay-Stammbach et al., 2014). Carlson (2003) argued that maternal parenting contributes to the development of EF through three primary aspects of interaction (scaffolding behavior, stimulation, and sensitivity) and that each has different effects on EF. *Scaffolding behavior*, which focuses on problem-solving, refers to how adults verbally and non-verbally guide and instruct children during challenging tasks (Lewis and Carpendale, 2009), support children’s autonomous exploration, and encourage children to make decisions autonomously to solve problems, (which also called autonomy support, Matte-Gagn and Bernier, 2011). For example, a mother might provide gentle guidance to a child frustrated by an ever-collapsing block tower by placing the larger blocks at the base. The strategies provided by caregivers tell children not only what to do, but also how to think, guiding improvements in cognitive control, error recognition, and self-correction during problem-solving. *Sensitivity* represents a caregiver’s ability to perceive and accurately interpret the signals shown explicitly or implicitly in an infant’s behavior, and to then respond appropriately (see Pederson et al., 1990). Children who experience their environments as predictable and consistent are inclined to achieve higher-level self-regulation in their early lives and more motivated to gain cognitive control (Carlson, 2003). For example, mother-toddler interaction with warm and responsive maternal parenting predicts longitudinal effortful control performance and delay of gratification ability (Kochanska et al., 2000; Sethi et al., 2000). *Stimulation*, as used here, covers the wide range of interactions between parents and children, such as the creation of environments for cognitive skill development through activities like reading (Bradley et al., 2011) and playing games together (Rome-Flanders et al., 1995). Maternal verbal input, which functions as a routine and important stimulus, has been found to play a critical role in children’s later executive processing (Landry et al., 2002) and may bolster the conversion to self-verbalization by which children gradually increase self-regulation in place of external regulation (Bernier et al., 2010). Maternal ‘mind-mindedness’ – the ability and tendency to nurture reflective functioning in the infant through interactive meaning-making – is inferred from caregivers’ tendencies to describe their children in terms of mental characteristics and to appropriately comment on their infants’ putative internal mental-related states (Meins et al., 2012). Compared to the quality of maternal sensitivity, mind-mindedness is characterized by not only recognizing baby’s request signals, such as crying due to hunger or gazing due to interest or the desire to play, but also echoing such requirements and explaining to the infant with appropriate and accurate oral responses (Meins et al., 2001). For example, when perceiving that a baby is hungry because he/she is crying, a sensitive mother will instantaneously feed the baby, but a mind-minded mother will soothe the baby with affective talk to manifest his/her requirement. During the interaction, the mother transmits emotion and affection to the baby and gives appropriate verbal feedback. Mind-mindedness has been consistently demonstrated to be linked to later expressive language in early toddlerhood and to EF (Bernier et al., 2012; Meins et al., 2012, 2013).

Bernier et al. (2010, 2012) studies have empirically examined the effects of these three aspects of maternal parenting (operationalized with sensitivity, autonomy support, mind-mindedness) of children aged 12–15 months on EF performance at 18, 26, and 36 months of age. Specifically, mind-mindedness and maternal autonomy support at 12–15 months predict EF at 18 months of age, whereas maternal autonomy support, and not sensitivity or mind-mindedness, is the exclusive predictor of conflict EF (including working memory and inhibitory control), but not impulse control EF (related to delay of gratification; Bernier et al., 2010), at 26 months. Another study of children’s EF performance at 38 months showed that maternal autonomy support has a consistently greater effect than mind-mindedness and sensitivity at 12–15 months of age (Bernier et al., 2012). In sum, studies conducted by Bernier et al. (2010, 2012) show that autonomy support at 12–15 months has more influence than mind-mindedness in predicting infants’ EF performance at 26 and 38 months.

However, relationships between these three aspects of maternal parenting during infants’ 1st year of life and later EF have not been examined. Several elements of maternal parenting of infants aged less than 1 year, expanding on prior studies that have focused on the infant’s 2nd year, should be considered. First, during the preverbal period, mothers are necessarily more focused on infant’s non-verbal behavior and bodily dependence during mother–infant interactions. Before they can verbally express their desires, mothers’ attunement to infants’ internal states (e.g., maternal mind-mindedness) and sensitive responses to infants’ demands may be more important during mother-infant interaction (Soderstrom, 2007; Meins, 2013). Additionally, infants aged < 1 year are less likely to engage in problem-solving requiring maternal scaffolding (Chen et al., 1997), which is more common in the 2nd year when infant’s exploration behavior and problem-solving orientated activities are more frequent. Thus, the effects of mind-mindedness and sensitivity are likely to be more conspicuous and consequential aspects of mother–infant interaction during the preverbal stage, while encouragement of autonomy may be more important in the 2nd year for predicting later development of EF as shown by Bernier et al. (2012). Secondly, according to Bernier et al. (2012), the relation of parenting and the development of EF may be mediated by attachment security, because the effect of parenting disappeared when those two variables are both entered into the regression model. In this case, due to the relatively large predictive power of mind-mindedness on attachment security compared to that of maternal sensitivity and autonomy support (e.g., Meins et al., 2001), mind-mindedness may be more important in the 1st year for EF’s development. Third, some research suggests cultural differences in the quality and effects of maternal parenting, specifically when Western and Chinese cultural settings are compared. Specifically, a cross-cultural research on mind-mindedness has shown that United Kingdom parents display higher levels of mind-mindedness than Hong Kong parents (Hughes et al., 2017). Such findings compel a close examination of early maternal parenting and the development of EF capacity.

The aim of the study reported here was to examine the effects of these three aspects of parenting (sensitivity, stimulation, scaffolding) during infants’ preverbal stage (age < 1 year) on EF at 2 and 3 years of age in a Chinese sample. In this study, these aspects were operationalized with maternal sensitivity, mind-mindedness, and encouragement of autonomy (Liu et al., 2009), which is comparable to the concept of autonomy support used by Bernier et al. (2010, 2012). For reasons outlined above, we expected that maternal mind-mindedness would be the most powerful predictors.

## Materials and Methods

### Participants

This study was part of a longitudinal research project that aimed to investigate the effects of early parenting environment on development outcomes, such as cognitive ability, social-emotional development, and language development. Dyads of mothers and infants aged 6 months were recruited by fliers or brought in by other participants in Beijing. Written parental informed consent was obtained. Data from dyads who did not participate in both assessments (at 25 and 38 months, *N* = 11) were not included in the final analysis.

### Analytic Sample

The initial sample consisted of 96 dyads of mothers and infants (42 males and 54 females) aged 6 [mean (*M*) = 6.46, standard deviation (*SD*) = 0.4, range = 5.6–7.4] months. Eleven dyads were excluded due to non-participation in both 25- and 38-month assessments. Excluded infants did not differ from those who participated (*n* = 85) with respect to sex [χ^2^ = 1.78, *p* = 0.16], age [*t*_(94)_ = 0.36, *p* = 0.724], family income [*t*_(89)_ = 0.73, *p* = 0.466], or parental education level [*t*_(78)_ = 0.65, *p* = 0.0.42]. In total, 76, 75, and 69 families participated in assessments when infants were aged 9 (*M* = 9.73, *SD* = 0.47, range = 9.3–10.7), 25 (*M* = 25.13, *SD* = 1, range = 23.8–27.3), and 38 (*M* = 38.2, *SD* = 0.77, range = 36.9–40.9) months, respectively. Family income was recorded with a 5-point scale: (1) *<18,000* (approximately $2,647*);* (2) *18,001–36,000 (approximately $2,648–$5,294)*; (3) *36,001–72,000 (approximately $5,295–$10,588)*; (4) *72,001–120,000 (approximately $10,589–$17,647)*; and (5) *>120,001(approximately $17,648) Chinese renminbi (RMB) per year.* Fathers’ and mothers’ average income scores were 3.76 (*SD* = 1.07) and 3.08 (*SD* = 0.99), respectively. Median income for both of mothers and fathers was between *36,000* and *72,000* RMB per year, approximating the average yearly income (*50,415* RMB, = approximately US$7,384 using 2010 conversion rates) in Beijing at that time (Beijing Municipal Bureau of Statistics, 2010). Most mothers (91.7%) and fathers (83.7%) had college-degree or higher education levels. Paternal and maternal educational levels were recorded as following categories: 1 = middle school educational level or below; 2 = high school educational level; 3 = college educational level; 4 = master educational level or above.

### Measures

#### General Cognitive Skill

Infant’s general cognitive skill was assessed by Mental Development Index (MDI) of the Bayley Scales of Infant Development II (Bayley, 2006). Although three semi-scales (the mental scale, psychomotor scale, and infant behavior record) were all measured at 6 months, the mental scale was always used to evaluate infant’s intelligence or general cognitive skill, thus in this study we only reported the infant’s MDI which is obtained via the mental scale.

#### Maternal Parenting Variables

*Sensitivity* was assessed at 9 months using the maternal behavior Q-sort (MBQS), a 72-item measure of maternal behavior during in-home mother–infant interactions (Pederson et al., 2009, unpublished). One research assistant completed the MBQS based on videotape review of 15 min mother–infant interaction. Seventy-two items describing potential maternal behaviors were sorted into nine categories, with scores gradually ranging from “very unlike (1)” to “very similar (9)” to the observed mothers’ behaviors, each category containing eight items. For example, item “responds immediately to cries/whimpers” which is very similar to the observed mothers’ behaviors should score 9, and items “when the baby is distressed, the mother is able to identify the source” which is very unlike to the observed mother’s behaviors should score 1. The scores of these items represent the sensitivity level of maternal caregiving behavior in a family setting. Then, the observer’s score was correlated with a standard score from an extremely sensitive mother, provided by the developers of the instrument (Pederson et al., 2009, Unpublished). MBSQ scores thus range from -1 (least sensitive) to 1 (extremely sensitive). Two research assistants rated the same 20 videotapes to obtain code consistency. The intercoder reliability was 0.76, *p* < 0.01. The measure has been shown to be reliable and valid in previous studies in Chinese children (e.g., *r* = 0.76 in Liang et al., 2015; *r* = 0.72 in Xing et al., 2016).

*Mind-mindedness* was coded using procedures adapted from Meins and Fernyhough (2006, Unpublished). Mothers’ speech during the sessions was transcribed verbatim, and all comments including internal state terms referring to the infants’ minds or emotions (mind-related comments) were identified from the transcripts. Five aspects were included in mind-related comments: (1) infant mental states, such as thoughts, knowledge, and desire (e.g., “do you want to read this book?”); (2) mental processes, included thinking, remembering, and knowing [e.g., “do you remember seeing a camel at the zoo?” (while the child plays with a toy camel)]; (3) emotions, such as shyness, solemnity, happiness, sadness, joy, and good/bad mood; (4) epistemic states, referring to teasing, playing games, and joking (e.g., “Are you playing games with me?”); and (5) talking on the infant’s behalf, referring to any utterance that is obviously meant to be said/thought by the infant (e.g., “you say we drink water!”). According to the coding manual (Meins and Fernyhough, 2006, Unpublished), mind-related comments were classified as appropriate or non-attuned, and only appropriate mind-related comments were used in this study to index mind-mindedness, expressed as the proportion of the total number of comments produced by a given caregiver during the interaction to control for differences in verbosity. Scores ranged from 0 to 1. The kappa value for the consistency of two research assistants’ coding of the same 21 videos was 0.817.

*Maternal encouragement of autonomy* was assessed using a coding manual developed by Liu et al. (2009). Verbal and non-verbal maternal behaviors that promoted children’s initiation of exploration self-directed exploration activities were assessed using five constructs: (1) giving suggestions, referring to mothers’ active provision of general suggestions and directives (e.g., “would you like to play with the bunny by yourself?” while pointing to the toy on the floor); (2) explanation, referring to mothers’ active and patient explanations of children’s behavior in detail (e.g., “baby, play like this, first, you can open….” while demonstrating); (3) providing selection, in which mothers provide several activities to children, who select desired activities (e.g., “Wow, so many toys there, which one will you want to play with first?” when mother and child enter the room); (4) positive reinforcement, including encouragement and praise (e.g., “well, you just did a good job”) for autonomous behaviors; and (5) direct commands given without force, referring to mothers’ giving of orders to children clearly and directly, with no emotional expression (e.g., “that’s amazing, keep going!” while the child is playing with a dump truck). Maternal encouragement of autonomous behavior was coded using an event-sampling approach with the scoring of the frequencies of maternal behaviors. Two research assistants performed coding for 20 randomly selected dyads (approximately 15% of the sample), and the kappa value for the inter-rater agreement was 0.803.

#### Executive Function

##### 25 months

*The Spin the Pots* task (Hughes and Ensor, 2005), used to assess children’s working memory at 25 months. Children were asked to search for six candies hidden randomly in eight opaque pots, two of which were left empty. The pots had very different visual appearances. The experimenter showed each candy when she put it into a pot and asked the child to remember which pots had candy. All pots were then covered with a piece of cloth and spun to change their locations. Then, the child was asked to retrieve the candy from the pots based on his/her memory. Only one pot could be opened at a time, and candy that had been found was placed on a table close to the child. The now empty pot was then put back in place, all the pots were covered with the cloth and spun again, and the exercise repeated. Three practice trials were conducted to ensure that the child understood what was expected followed by a maximum of 16 test trials to find as many candies as possible [based on Hughes and Ensor (2005) approach]. Scores were computed by subtracting the number of incorrect finds from the total of 16 trials and theoretically ranged from 0 to 16.

*Reverse categorization* (Carlson et al., 2004). This task was tapped to measure toddler’s inhibition ability. Children were presented with six larger blocks, six smaller blocks, and two boxes and were asked to help the experimenter collect the large blocks into the “big” box and the small blocks into the “small” box. Then, the experimenter suggested that they play a different game with the reverse categorization rule (small blocks in the “big” box and large blocks in the “small” box). The experimenter presented the 12 blocks randomly, one by one, with a reminder about the rule at each presentation. The score (0–12) represented the number of blocks correctly placed by the child.

*Externally imposed delay* [adapted from Kochanska et al. (2000)], which is commonly used to measure externally imposed delay ability. An open transparent box containing an attractive toy tiger was introduced to the child. The toy tiger had a switch and could make a sound and sing a song when turned on. The experimenter told the child that she was going outside to get something, and asked him/her to not touch the toy before she came back. Upon the child’s indication of understanding with a nod or other such behavior, the experimenter left the room. The mother could stay in the room, but only watch; she was not allowed to provide hints with vocal or non-vocal behavior. The trial was terminated when child touched the tiger or the box, or 3 min after the experimenter had left. Two indexes were calculated for the evaluation of children’s externally imposed delay ability: the waiting time (maximum 180 s) and the extent of behavior [(1) removing the tiger from the box and playing with it; (2) touching the tiger without removing it from the box; (3) touching the box only; (4) not touching the tiger or the box]. Given the high correlation between these indexes (*r* = 0.871, *p* < 0.001), a composite score was computed by averaging the standardized scores of both indexes.

##### 38 months

*Working memory span-like task* [adapted from Willoughby et al. (2010)]. This task was conducted to assess children’s working memory at 38 months. Children were presented with a picture of a house, with an animal drawing at the bottom of the house and a colored dot at the top of the house. In the warm-up phase, the experimenter asked the child to name the color and the animal. Then, the experimenter turned to a page that showed only the outline of the house depicted on the previous page and asked the child to recollect which animal was in the house on the previous page. This task targeted children’s working memory ability, which requires them to hold in mind two pieces of information (i.e., the animal’s identity and the dot’s color) simultaneously and to actively name the animal while overcoming interference from naming the dot’s color. Following the approach used by Willoughby et al. (2010), only 11 items were administered at 38 months assessment. The task comprised three trial types, performed in the following order: one one-house trial, two two-house trials, and two three-house trials. Each item was scored as 1 (recall of all animals) or 0 (no/incomplete recall of animals). Scores were indexed with the number of correct-response items and theoretically ranged from 0 to 11.

*Day*–*night* [adapted from Carlson (2005)]. This task is used commonly to assess children’s inhibitory ability, as it requires them to activate a subdominant response while inhibiting the dominant behavior. In the brief warm-up, the child was presented with two drawings (of a yellow sun and a black moon), and the experimenter talked with him/her about when the sun and moon appear (in the day and night, respectively). The experimenter guided the child to say “day” when seeing the sun picture and “night” when seeing the moon picture. In the formal performance phase, the child was instructed to respond inversely to the convention, saying “night” when presented with the sun picture and “day” when presented with the moon picture. This phase comprised 16 trials. A correct response in each trial was scored as 1, resulting in a total score ranging from 0 to 16.

*Gift delay* [adapted from Kochanska et al. (2000)]. The child was informed that he/she would get a reward for his/her good performance, but that the experimenter needed to wrap the gift first. He/she was asked to sit with his/her back to the experimenter and to not peek while the gift was being wrapped (duration, 60 s). The experimenter deliberately made sounds of the gift to attract the child while wrapping it. Scores for the extent of peeking or turning ranged from 1 (turns around and continues to peek) to 3 (peeks over shoulder) to 5 (does not peek), and the latency to peeking or turning was also recorded (60 s if the child never peeked or turned). Given the high correlation between these indexes (*r* = 0.80, *p* < 0.001), a composite score was computed by averaging the standardized scores of both indexes.

*Self-imposed delay* [adapted from Kochanska et al. (2000)]. After wrapping the gift for the gift delay task, the experimenter asked the child to face her. The experimenter placed the wrapped gift on a table and told the child that she would leave the room to get something and that the child was not allowed to touch the gift until she returned (180 s later). The experimenter also told the child that if he/she touched the gift before the experimenter returned, he/she would be allowed to play with the gift for only a moment in the laboratory; if he/she did not touch the gift, he/she could take it home as a real gift. The child’s wait time was recorded and served as the score.

### Procedure

At baseline (children aged 6 months), demographic data (including father’s and mother’s educational level and annual income) were collected via questionnaire administered to mothers during home visits, and general cognitive development was assessed using the MDI of the Bayley Scales of Infant Development II (Bayley, 2006) in the university laboratory. These variables assessed at age 6 months were regarded as control variables to predict the development of later EF.

The second home visit (when children were aged 9 months) was designed to observe mother–infant interaction using three semi-structured procedures in which age-appropriate toys were available for a mother–infant play sequence: a cloth picture book (for mother–infant book-sharing), beads (which mothers could direct infants to string on a cord), and blocks (with which mothers could instruct infants to build shapes). Each toy was available for a 5-min period. All procedures were videotaped using two cameras focused on the mother and infant, respectively. Three maternal parenting aspects (Carlson, 2003) were coded from these videotapes: maternal sensitivity and mind-mindedness were coded during all three procedures during a 15-min period, and maternal encouragement of autonomous behavior was coded during the bead and block sessions during a 10-min period (due to the low frequency of autonomous behavior during the cloth book procedure).

At the third assessment (when children were aged 25 months), three EF tasks were conducted in the laboratory; in order, these were pot spinning (working memory), reverse categorization (inhibitory control) and an externally imposed delay task (delay EF).

Similarly, at the final assessment (when children were aged about 38 months), four age-appropriate EF tasks were conducted in the laboratory: in order, these were working memory span (working memory), day–night (inhibitory control), gift delay (delay EF), and self-delay tasks (delay EF). Following Bernier et al. (2012), assessment of children’s EF at 2 and 3 years in this study focused on three dimensions: working memory, inhibitory control, and delay EF ability. The corresponding tasks have been proved valid and reliable to measure these components (see Kochanska et al., 2000; Carlson et al., 2004; Willoughby et al., 2010). We explored externally imposed delay, such as at parents’ requests (Mauro and Harris, 2000), at 2 years of age, as it is more pervasive at this age and has been used for the assessment of delay EF ability (Kochanska et al., 2000). We assessed gift delay and self-imposed delay (which reflects children’s self-regulation ability in a competitive stress context; Mischel et al., 1988) at 3 years of age.

### Statistical Analysis

Given the moderate correlations between maternal and paternal income (*r* = 0.593, *p* < 0.01) and the concision of these regression models (8 or 9 variables already entered in the model but our sample was just 85), according to the method used in Bernier et al. (2012), these variables were standardized and averaged into a composite index of family income. Due to the modest correlations between maternal education and parental education (*r* = 0.323, *p* < 0.01), these two variables severed as covariates separately in the following analysis.

First, the correlation analysis was conducted to test the coherence in three aspects of maternal parenting quality, the internal consistency of those three EF constructs at either time point, and the individual longitudinal stability of EF constructs between 25 and 38 months. Next, hierarchical regression analysis was conducted to evaluate the ability of maternal parenting aspects at 9 months to predict EF constructs (working memory, inhibitory control, and delay EF) at 25 and 38 months. For predicting EF at 25 months, the first block included the covariates gender, maternal and parental education level, family income, and MDI, and the second block contained maternal parenting constructs. For predicting EF at 38 months, the first block variables were similar with that at 25 months, the corresponding EF construct tested at 25 months formed the second block of the model, and maternal parenting aspects were entered in the third block.

### Missing Data

The percentages of missing data (12–30%) for these study variables are presented in **Table 1**. Little’s test (Little, 1988) was applied to assess the influence of missing data. The results indicated that the pattern of missing data was non-systematic [χ^2^_(362)_ = 36.74, *p* = 0.509], supporting the use of missing data imputation. According to Collins et al. (2001), we applied expectation maximization to impute the missing data to yield optimal results. It should be noted that the following analyses are based on imputed data only, and the results hold when using the raw data set.

**Table 1:** Descriptive statistics for parenting and executive function measures.

	Missing data rate	*M* (*SD*)	Observed range	Theoretical range
Maternal education	0%	3.27 (0.59)	2–4	1–5
Parental education	10.6%	3.24 (0.71)	2–4	1–5
Family income	2.4%	0.04 (1.78)	-4.05–3.09	N/A
MDI 6 months	0%	98.8 (5.79)	84–113	N/A
Maternal parenting 9 months				
Mind-mindedness	14%	0.04 (0.03)	0–0.12	0–1
Encouragement of autonomy	18%	3.46 (11.74)	6.87–72.79	0–N/A
Sensitivity	18%	0.59 (0.14)	0.09–0.84	-1–1
EF 25 months				
Spin the pots (WM)	21%	8.63 (2.73)	4–15	0–16
Reverse categorization (IC)	15%	7.50 (4.23)	0–12	0–12
Externally imposed delay	16%	0.01 (1.77)	-2.33–2.95	N/A
EF 38 months				
WM span (WM)	27%	6.08 (1.82)	1–11	0–11
Day/night (IC)	30%	10.08 (4.17)	0–16	0–16
Gift delay	25%	-0.03 (1.69)	-4.17–2.37	N/A
Self-imposed delay	24%	107 (68.45)	0–180	0–180

*M, mean; SD, standard deviation; MDI, Bayley Mental Development Index; EF, executive function; WM, working memory; IC, inhibitory control*.

## Results

### Descriptive Statistics and Correlations

Descriptive statistics and missing data rates for all study variables are presented in **Table 1**. The mean score of mind-mindedness was 0.04, suggests that there was just a small proportion of appropriate mental-related comments during the interaction. Bivariate correlations between all variables are presented in **Table 2**. Maternal parenting constructs at 9 months were unrelated to each other. Little coherence across EF tasks was exhibited at 25 months because the associations across these EF tasks were not significant (*r*s < 0.10, *p*s > 0.35). At 38 months, there was much more coherence across EF tasks, as working memory was related positively to inhibitory control (*r* = 0.27, *p* < 0.05), but no other variable; gift delay showed modest associations with inhibitory control (*r* = 0.25, *p* < 0.05) and self-imposed delay (*r* = 0.31, *p* < 0.01). Measures of EF constructs obtained across time points were not related, demonstrating low levels of stability of EF constructs from 25 to 38 months. Finally, maternal parenting aspects were associated positively with certain EF constructs. Mind-mindedness was related significantly to inhibitory control at 25 months (*r* = 0.39, *p* < 0.01) and 38 months (*r* = 0.30, *p* < 0.01) and to gift delay at 38 months (*r* = 0.27, *p* < 0.05). Encouragement of autonomy was associated positively with inhibitory control (*r* = 0.22), gift delay (*r* = 0.25), and self-imposed delay (*r* = 0.24) at 38 months (all *p* < 0.05).

**Table 2:** Correlation matrix for all variables (*n* = 85).

		1	2	3	4	5	6	7	8	9	10	11	12	13	14	15
	**9 months**															
(1)	Mind-mindedness	1														
(2)	Encouragement of autonomy	0.06	1													
(3)	Sensitivity	0.06	0.06	1												
	**25 months**															
(4)	Working memory	0.17	-0.01	-0.18	1											
(5)	Inhibitory control	0.39**	0.07	0.15	0.10	1										
(6)	Externally imposed delay	-0.15	0.06	-0.01	-0.10	-0.03	1									
	**38 months**															
(7)	Working memory	0.03	0.13	-0.08	0.09	0.07	-0.04	1								
(8)	Inhibitory control	0.30**	0.22*	0.02	0.06	0.05	-0.30**	0.27*	1							
(9)	Gift delay	0.27*	0.25*	-0.13	0.03	0.08	-0.14	0.04	0.25*	1						
(10)	Self-imposed delay	-0.02	0.24*	-0.03	0.21	-0.18	-0.17	0.10	0.07	0.31**	1					
	**Covariates**															
(11)	Gender^a^	-0.05	0.13	-0.06	0.01	0.10	-0.23*	0.21*	0.04	0.25*	0.23*	1				
(12)	Maternal education	0.15	0.01	0.05	-0.23*	0.07	-0.05	0.28**	0.22*	0.13	-0.07	0.11	1			
(13)	Parental education	0.10	-0.18	-0.06	-0.17	-0.08	0.17	0.04	-0.13	0.19	-0.04	-0.05	0.33**	1		
(14)	Family income	0.10	-0.13	-0.03	-0.12	0.10	-0.12	0.09	0.09	0.18	-0.05	0.01	0.24*	0.31**	1	
(15)	MDI	0.03	-0.20	0.02	0.21	-0.13	-0.01	0.10	-0.04	0.05	0.41**	0.07	-0.04	-0.11	-0.01	1

^a^*Dummy variable (0 = boy, 1 = girl); MDI, Bayley Mental Development Index; EF, executive function. ^∗^*p* < 0.05, ^∗∗^*p* < 0.01*.

### Predictive Effects of Maternal Parenting on Executive Function at 25 Months

**Table 3** shows the extent to which mind-mindedness, encouragement of autonomy, and sensitivity in infancy predict working memory, inhibitory control, and delay EF at 25 months when controlling for covariates. The first model yielded no significant result, and accounted for 6–11% of the variance in the three EF constructs [*F*s(5,79) < 2.03, *ns*]. Only gender was found to be negatively correlated with delay EF at 25 months (β = -0.26, *p* < 0.05).

**Table 3:** Summary Regression results for the prediction of executive function at 25 months.

		Working memory			Inhibitory control			Delay EF		
		Δ*R*^2^	Δ*F*	β	Δ*R*^2^	Δ*F*	β	Δ*R*^2^	Δ*F*	β
(1)	Gender^a^	0.10	1.80	-0.02	0.06	0.95	0.13	0.11	2.03	-0.26*
	Maternal education			-0.20			0.02			0.08
	Parental education			-0.10			-0.17			0.23
	Family income			-0.07			0.10			-0.17
	MDI			0.19			-0.17			0.08
(2)	Mind-mindedness	0.08	2.51*	0.23*	0.17	5.72**	0.40**	0.05	1.49	-0.20
	Encouragement of autonomy			0.003			-0.04			0.14
	Sensitivity			-0.19			0.14			-0.01

^a^*Dummy variable (0 = boy, 1 = girl). MDI, Bayley Mental Development Index; EF, executive function. ^∗^*p* < 0.05, ^∗∗^*p* < 0.01. β is for the final model*.

In the last model, maternal parenting constructs uniquely explained 8% of the variance in working memory [Δ*F*(3,76) = 2.13, *p* < 0.05], 17% of the variance in inhibitory control [Δ*F*(3,76) = 2.85, *p* < 0.01], and 5% of the variance in delay EF task scores [Δ*F*(3,76) = 1.85, *p* = 0.08] at 25 months. More specially, among the maternal parenting constructs, mind-mindedness was the only predictor of working memory (β = 0.23, *p* < 0.05) and inhibitory control (β = 0.40, *p* < 0.01) but not of delay EF (β = -0.20, *p* = 0.07) at 25 months. Neither encouragement of autonomy nor sensitivity were related to any aspect of EF at 25 months (*p*_s_ > 0.07).

### Predictive Effects of Maternal Parenting on Executive Function at 38 Months

**Table 4** summarizes the results of regression analysis testing the ability of maternal parenting constructs to predict EF at 38 months. The first (covariate) model accounted for 12, 11, 12, and 22% of the variance in working memory, inhibition control, gift delay, and self-delay, respectively. But only the model of self-delay significantly accounted for the variance [*F*(1,78) > 4.37, *p* < 0.01]. Additionally, for the effects of covariates, only maternal education was related with working memory (β = 0.29, *p* < 0.05) and MDI was related with self-delay (β = 0.49, *p* < 0.01) at 38 months. In the second model, only the EF delay at 25 months were negatively related with self-delay at 38 months (β = -0.21, *p* < 0.05), while the other EF constructs at 25-months were not significantly accounted for the variance of the corresponding EF constructs at 38 months (*p*s > 0.14).

**Table 4:** Summary regression results for the prediction of executive function at 38 months.

		Working memory			Inhibitory control			Gift delay			Self-delay		
		Δ*R*^2^	Δ*F*	β	Δ*R*^2^	Δ*F*	β	Δ*R*^2^	Δ*F*	β	Δ*R*^2^	Δ*F*	β
(1)	Gender^a^	0.12	2.21	0.14	0.11	1.88	0.01	0.12	2.20	0.19	0.22	4.37**	0.11
	Maternal education			0.29*			0.23			-0.004			-0.08
	Parental education			-0.01			-0.27			0.22			0.16
	Family income			0.06			0.12			0.12			-0.05
	MDI			0.11			-0.05			0.12			0.49**
(2)	Working memory 25 months	0.02	2.12	0.13									
	Inhibitory control 25 months				0.002	0.01	-0.14						
	Delay EF 25 months							0.01	2.00	-0.10	0.02	2.19	-0.21*
(3)	Mind-mindedness	0.02	1.64	-0.04	0.12	3.95*	0.33**	0.15	5.13**	0.22*	0.11	4.31**	-0.05
	Encouragement of autonomy			0.15			0.17			0.30**			0.36**
	Sensitivity			-0.07			-0.01			-0.14			-0.04

^a^*Dummy variable (0 = boy, 1 = girl), MDI, Bayley Mental Development Index; EF, executive function. ^∗^*p* < 0.05, ^∗∗^*p* < 0.01. β is for the final model*.

In the third model, maternal parenting aspects explained an additional 12% of the variance in inhibitory control [Δ*F*(3,75) = 3.95, *p* < 0.05], 15% of the variance in gift delay [Δ*F*(3,75) = 5.13, *p* < 0.01], and 11% of the variance in self-delay [Δ*F*(3,75) = 4.31, *p* < 0.01]. But only 2% of the variance in working memory [Δ*F*(3,75) = 1.64, *ns*]. The performance of maternal parenting aspects in predicting EF constructs at 38 months varied. For working memory, none of the three maternal parenting constructs predicted working memory at 38 months (*p*_s_ > 0.19). For inhibitory control, only mind-mindedness positively predicted inhibitory control (β = 0.33, *p* < 0.01), encouragement of autonomy and sensitivity were not predictive (*p*_s_ > 0.13). For delay EF, mind-mindedness and encouragement of autonomy positively predicted gift delay (respectively, β = 0.22, *p* < 0.05; β = 0.36, *p* < 0.01), encouragement of autonomy positively predicted self-delay (β = 0.30, *p* < 0.01), sensitivity was not related with both of delay EF tasks (*p*_s_ > 0.18). In sum, mind-mindedness were predictive of inhibitory control, encouragement of autonomy was predictive of delay EF, and sensitivity were not related with these EF constructs.

## Discussion

The current study aimed to examine the contributions of maternal parenting environments in infants’ 1st year to EF at 2 and 3 years of age. We found that maternal mind-mindedness when infants are aged 9 months predicted inhibitory control when they reach 2 and 3 years. In addition, in line with our expectations, maternal encouragement of autonomous behavior predicted good performance on delay EF tasks at 3 years, whereas maternal sensitivity had no observable effect on children’s EF. These results contrast with those reported by Bernier et al. (2010, 2012), which showed that maternal autonomy support 2 years was the most predictive factor for conflict EF 1 and 2 years later.

Before discussing the effects of maternal parenting aspects on later EF, it should be noted that – while validated measures were used – the three constructs of maternal parenting were not inter-correlated in the 1st year, and coherence across these EF constructs was not found at 25 months. For maternal parenting, the uncorrelated results are counter to findings from Bernier et al. (2012), which showed moderate relationships among the three constructs in the 2nd year. There are three possible explanations for this inconsistency. First, the uncorrelated results among the three constructs in the 1st year in our sample may suggests that mothers have not formed a consistent parenting style during the 1st year when they are new mothers. For example, Meins et al. (2001) have reported no significant correlations between mother’s encouragement of autonomy and maternal sensitivity (*r* = 0.09, *ns*) or mind-mindedness (*r* = -0.03, *ns*) at 6 months. Second, this may be also due to the culture-based difference in parenting practice. For example, one study has shown that mothers in Hong Kong demonstrate less mind-mindedness than United Kingdom mothers (Hughes et al., 2017). We also found that mind-mindedness was demonstrated less frequently by Chinese mothers in this study compared to among Canadian mothers in Bernier et al. (2010)’s work (in this study, *M* = 5.56, *SD* = 4.30; in Bernier’s work, *M* = 14.58, *SD* = 8.71, *t* = 6.59, *p* < 0.01). Third, these uncorrelated relations may be explained with the perspective of domain-specificity on children’s socialization. According to Grusec and Davidov (2010), there are five domains in the process of socialization: protection, reciprocity, control, guided learning, group participation. These parenting constructs may correspond to a different domain, with sensitivity referring to a protection domain, mind-mindedness referring to reciprocity domain, and encouragement of autonomy referring to guided learning domain. These different domains focus on different parenting goals, and thus are not correlated in the 1st year of life.

Considering the development of EF in this study, the results showed that the coherence at 25 months is very low, with higher consistency across those EF tasks at 38 months, consistent with the results found by Miller and Marcovitch (2015). Miller and Marcovitch (2015) have suggested that in the 2nd year, children display very little coherence or stability across a battery of EF tasks. Our results are consistent with this pattern and extend it to suggest that there is little stability across EF tasks from 25 to 38 months. In sum, the little coherence in the development of EF during the first 3 years infers the EF constructs may differentiate in the early of life. It should be also noted that the first 3 years always be recognized as a period of significant growth, multiple timepoint measures may help to display the development of EF during the early of life.

In contrast with the moderate association of mind-mindedness with EF, we found no predictive effect of maternal sensitivity on EF development at 2 or 3 years of age, a pattern consistent with Bernier et al.’s (2010) study which found sensitivity to be relatively weakly associated with EF. In the 1990s, maternal sensitivity was found to be the core index of the quality of mother–infant interaction and the most efficient predictor of mother–infant attachment. In recent decades, however, Meins et al. (2001), Meins (2013) have argued that mind-mindedness is a more efficient predictor of such attachment. Tomlinson et al. (2005) also suggest that maternal sensitivity (including responsiveness, acceptance, and warmth) at 2 months was no longer predictive for attachment at 18 months when considering maternal intrusiveness and remoteness. Thus, the results of the current study emphasize that children should be regarded as mental agents and mothers should be attuned to children’s mental states and explain to them appropriately, rather than simply perceiving their needs and providing material support. More importantly, Bernier et al. (2012) reported that the effect of maternal parenting is attenuated when it is considered jointly with that of mother–infant attachment in predicting EF in early childhood. That is, maternal parenting may impact the development of EF via mother–infant attachment. Thus, in the 1st year, mind-mindedness may be more effective to predict EF than maternal sensitivity (Meins et al., 2012).

Moreover, maternal mind-mindedness refers to the tendency to view the child as a mental agent, particularly in the infant’s 1st years (Meins et al., 2001, 2012). Mothers’ appropriate utterance of infants’ thoughts/cognitions/emotions promotes infants’ internal language representation to the external environment. With internal language processing, infants can complete difficult and complex cognitive tasks effectively. For example, children strengthen and extract the rules of the day–night task via internal language, saying “day” when seeing the moon and “night” when seeing the sun. Mind-mindedness during infants’ 1st year may be a more effective predictor than the same aspect in the 2nd year of later representational ability (Meins et al., 2013), which in turn can predict EF development 2 years later (Miller and Marcovitch, 2015).

In addition, with consideration of the evidence provided by Bernier et al. (2010, 2012) suggesting that autonomy support is a stronger predictive factor than mind-mindedness for EF development in infants’ 2nd year, mind-mindedness may be more important for EF development in the 1st year, whereas encouragement of autonomy may be more important in the 2nd year, during which infants need more instructions on how to interact with the environment. It should be noted that although the concepts of encouragement of autonomy and maternal autonomy support are similar, it may be that subtle differences between these contribute to inconsistencies between this study and prior studies. For future research, the contributions of maternal parenting during infants’ 1st and 2nd year on later EF development should be considered within a single sample.

Although we found that maternal mind-mindedness played a more important role in the development of EF, we also found that maternal encouragement of autonomy in infants’ 1st year had a predictive effect mainly on the delay of gratification ability at the age of 3 years. These findings are consistent with those of Matte-Gagn and Bernier (2011), who reported that infants who experience greater autonomy support at 15 months have better verbal skills at 2 years, and thus better performance on impulse control EF tasks (delay of gratification) at 3 years; no effect was observed, however, on working memory or inhibitory control. In the delay of gratification task, children must use internal self-regulation, choosing either peek and touch in temporary play or not peek and wait to receive the toy. Autonomy support and encouragement of autonomy prompt children’s internal self-regulation, which results in children making more rational decisions and solving problems more efficiently. However, the three aspects of maternal parenting examined in this study were not predictive of delay EF ability at 25 months of age. The difference in delay EF tasks given to children at 2 and 3 years of age should be considered. With the externally imposed task, applied at 2 years of age, children should conform to the experimenter’s instruction and inhibit their curiosity (not touch the toy before the experimenter returns) (Mauro and Harris, 2000). With the self-imposed delay task, applied at 3 years of age, children need to estimate self-regulating rules and strategies by themselves (deciding independently whether to play with the toy for a while or take it home) (Mischel and Ebbesen, 1970; Kochanska et al., 2000). Our findings suggest that relative to the self-imposed delay task, maternal parenting did not prompt the development of externally imposed delay ability.

In addition, we found a discrepancy in the relation of maternal parenting to EF tasks related to working memory and inhibitory control. Unlike previous studies, which have examined two dimensions of EF (conflict and delay EF), we considered EF constructs separately and found that maternal parenting was not related positively to working memory at 2 or 3 years of age. Although we used only one task with proven validity (e.g., Hughes and Ensor, 2005; Willoughby et al., 2011) to assess working memory at 25 and 38 months, the similarity of results at both time points suggest that the effects of maternal parenting on working memory may not be as strong as previously thought. Our results are in accordance with results of a previous study that shows no positive relationship between maternal scaffolding and space-related working memory (Hammond et al., 2012). Working memory differs from inhibitory control in that it requires only the maintenance and appropriate manipulation of information, whereas inhibitory control requires children to inhibit the dominant response and to solve problems using new rules (Diamond, 2013). Maternal parenting may encourage children to inhibit the dominant response and rebuild rule structures, rather than just helping them to maintain information in the mind. In this study, the MDI was correlated positively with working memory ability at 2 years of age, which may primarily indicate that the ability to maintain information (reflected by working memory) is related to general cognition (reflected by the MDI). Further studies should examine the mechanism accounting for this difference in the relationships of maternal parenting to working memory and inhibitory control.

### Limitations

The results of the present study, conducted with a Chinese sample, suggest that mind-mindedness and encouragement of autonomy at infants’ age of 9 months are predictive of EF development at 3 years, with the former being more predictive of inhibitory control and the latter being more predictive of delay EF ability. Although we report new evidence for the effect of maternal parenting during infants’ 1st year on later EF development, the results must be interpreted carefully.

First, although this study has a conclusion that mind-mindedness is more important in the 1st years to the EF development at 2 and 3 years, with no intensive observation at multiple time points, we could not identify aspects of maternal parenting that are most important for EF development during the entire childhood period. As the correlation analysis in this study showed that parenting behavior was not as stable as expected, and its effects on outcomes in children vary across early childhood, future research should include more intensive measure time points and assess both maternal parenting aspects and EF at each time.

Second, although the discrepancies between the results of this study and those of previous studies may be due to the differences in effects of maternal parenting in Western and Chinese cultures, they cannot be explained entirely by cultural factors. Differences in experimental materials and procedures may also have effects, and this study was not cross-cultural. Thus, future research should include cross-cultural and longitudinal elements to properly examine related effects of parenting on children’s EF development.

## Conclusion

In sum, despite its limitations, this study is the first, to our knowledge, to examine the predictive effect of maternal parenting quality at 9 months on EF task performance at 2 and 3 years of age in a Chinese sample. The results suggest that mind-mindedness is the strongest predictive factor for inhibitory control when children are 2 and 3 years of age and that encouragement of autonomy is the strongest predictor of delay of gratification ability when children were 3 years of age.

## Ethics Statement

This study was carried out in accordance with the recommendations of Ethics Committee of Capital Normal University with written informed consent from all subjects. All subjects gave written informed consent in accordance with the Declaration of Helsinki. The protocol was approved by the Ethics Committee of Capital Normal University.

## Author Contributions

SL designed the experiment. ZW planned and supervised the process of this project. NC conducted the experiment and analyzed the data, drafted the manuscript. SL and MA have been involved the revision of this manuscript.

## Conflict of Interest Statement

The authors declare that the research was conducted in the absence of any commercial or financial relationships that could be construed as a potential conflict of interest.

## References

[B1] Bayley N. (2006). *Bayley Scales of Infant and Toddler Development: Bayley-III.* San Antonio, TX: Harcourt Assessment Inc.

[B2] Beijing Municipal Bureau of Statistics (2010). *Beijing Statistical Yearbook.* Beijing: China Statistical Publishing House.

[B3] Benson J. E. Sabbagh M. A. Carlson S. M. Zelazo P. D. (2013). Individual differences in executive functioning predict preschoolers’ improvement from theory-of-mind training. *Dev. Psychol.* 49 1615–1627. 10.1037/a0031056 23244411

[B4] Bernier A. Carlson S. M. Deschênes M. Matte-Gagné C. (2012). Social factors in the development of early executive functioning: a closer look at the caregiving environment. *Dev. Sci.* 15 12–24. 10.1111/j.1467-7687.2011.01093.x 22251288

[B5] Bernier A. Carlson S. M. Whipple N. (2010). From external regulation to self-regulation: early parenting precursors of young children’s executive functioning. *Child Dev.* 81 326–339. 10.1111/j.1467-8624.2009.01397.x 20331670

[B6] Bradley R. H. Mckelvey L. M. Whiteside Mansell L. (2011). Does the quality of stimulation and support in the home environment moderate the effect of early education programs? *Child Dev*. 82 2110–2122. 10.1111/j.1467-8624.2011.01659.x 22004351

[B7] Bull R. Lee K. (2014). Executive functioning and mathematics achievement. *Child Dev. Perspect.* 8 36–41. 10.1111/cdep.12059

[B8] Carlson S. M. (2003). Executive function in context: development, measurement, theory, and experience. *Monogr. Soc. Res. Child Dev.* 68 138–151. 10.1111/j.0037-976X.2003.00270.x 14723274

[B9] Carlson S. M. (2005). Developmentally sensitive measures of executive function in preschool children. *Dev. Neuropsychol.* 28 595–616. 10.1207/s15326942dn2802-3 16144429

[B10] Carlson S. M. (2009). Social origins of executive function development. *New Dir. Child Adolesc. Dev.* 123 87–98. 10.1002/cd.237 19306276

[B11] Carlson S. M. Mandell D. J. Williams L. (2004). Executive function and theory of mind: stability and prediction from ages 2 to 3. *Dev. Psychol.* 40 1105–1122. 10.1037/0012-1649.40.6.1105 15535760

[B12] Chen Z. Sanchez R. P. Campbell T. (1997). From beyond to within their grasp: the rudiments of analogical problem-solving in 10- and 13-month-olds. *Dev. Psychol.* 33 790–801. 10.1037/0012-1649.33.5.790 9300212

[B13] Collins L. M. Kam C. Kam C. (2001). A comparison of restrictive strategies in modern missing data procedures. *Psychol. Methods* 6 330–351. 10.1037/1082-989X.6.4.330 11778676

[B14] Diamond A. (2013). Executive functions. *Annu. Rev. Psychol.* 64 135–168. 10.1146/annurev-psych-113011-143750 23020641 PMC4084861

[B15] Fay-Stammbach T. Hawes D. J. Meredith P. (2014). Parenting influences on executive function in early childhood: a review. *Child Dev. Perspect.* 8 258–264. 10.1111/cdep.12095

[B16] Garon N. Bryson S. E. Smith I. M. (2008). Executive function in preschoolers: a review using an integrative framework. *Psychol. Bull.* 134 31–36. 10.1037/0033-2909.134.1.31 18193994

[B17] Grusec J. E. Davidov M. (2010). Integrating different perspectives on socialization theory and research: a domain-specific approach. *Child Dev.* 81 687–709. 10.1111/j.1467-8624.2010.01426.x 20573097

[B18] Hammond S. I. Müller U. Carpendale J. I. M. Bibok M. B. Liebermann-Finestone D. P. (2012). The effects of parental scaffolding on preschoolers’ executive function. *Dev. Psychol.* 48 271–281. 10.1037/a0025519 21928877

[B19] Hendry A. Jones E. J. H. Charman T. (2016). Executive function in the first three years of life: precursors, predictors and patterns. *Dev. Rev.* 42 1–33. 10.1016/j.dr.2016.06.005

[B20] Hughes C. Devine R. T. Wang Z. (2017). Does parental Mind-Mindedness account for Cross-Cultural differences in preschoolers’ theory of mind. *Child Dev.* 10.1111/cdev.12746 [Epub ahead of print]. 28160284 PMC6849528

[B21] Hughes C. Ensor R. (2005). Executive function and theory of mind in 2-year-olds: a family affair? *Dev. Neuropsychol*. 28 645–668. 10.1207/s15326942dn2802-5 16144431

[B22] Hughes C. H. Ensor R. A. (2009). How do families help or hinder the emergence of early executive function? *New Dir. Child Adolesc. Dev*. 123 35–50. 10.1002/cd.234 19306273

[B23] Kochanska G. Murray K. T. Harlan E. T. (2000). Effortful control in early childhood: continuity and change, antecedents, and implications for social development. *Dev. Psychol.* 36 220–232. 10.1037/0012-1649.36.2.220 10749079

[B24] Kok R. Lucassen N. Bakermans-Kranenburg M. J. van Ijzendoorn M. H. Ghassabian A. Roza S. J. (2014). Parenting, corpus callosum, and executive function in preschool children. *Child Neuropsychol.* 20 583–606. 10.1080/09297049.2013.832741 24028215

[B25] Kuhn L. J. Willoughby M. T. Wilbourn M. P. Vernon-Feagans L. Blair C. B. (2014). Early communicative gestures prospectively predict language development and executive function in early childhood. *Child Dev.* 85 1898–1914. 10.1111/cdev.12249 24773289 PMC4165687

[B26] Landry S. H. Miller-Loncar C. L. Smith K. E. Swank P. R. (2002). The role of early parenting in children’s development of executive processes. *Dev. Neuropsychol.* 21 15–41. 10.1207/S15326942DN2101-2 12058834

[B27] Lewis C. Carpendale J. I. M. (2009). Introduction: links between social interaction and executive function. *New Dir. Child Adolesc. Dev.* 123 1–15. 10.1002/cd.232 19306271

[B28] Liang X. Wang Z. Y. Liu H. Y. Lin Q. Wang Z. Liu Y. (2015). Adult attachment status predicts the developmental trajectory of maternal sensitivity in new motherhood among Chinese mothers. *Midwifery* 31 68–73. 10.1016/j.midw.2014.05.011 24986812

[B29] Little R. J. (1988). A test of missing completely at random for multivariate data with missing values. *J. Am. Stat. Ass.* 83 1198–1202. 10.1080/01621459.1988.10478722

[B30] Liu M. Chen X. Zheng S. (2009). Maternal autonomy- and connectedness-oriented parenting behaviors as predictors of children’s social behaviors in china. *Soc. Dev.* 18 671–689. 10.1111/j.1467-9507.2008.00501.x

[B31] Matte-Gagn C. Bernier A. (2011). Prospective relations between maternal autonomy support and child executive functioning: investigating the mediating role of child language ability. *J. Exp. Child Psychol.* 110 611–625. 10.1016/j.jecp.2011.06.006 21798554

[B32] Mauro C. F. Harris Y. R. (2000). The influence of maternal child-rearing attitudes and teaching behaviors on preschoolers’ delay of gratification. *J. Genet. Psychol.* 161 292–306. 10.1080/00221320009596712 10971908

[B33] Meins E. (2013). Sensitive attunement to infants’ internal states: operationalizing the construct of mind-mindedness. *Attach. Hum. Dev.* 15 524–544. 10.1080/14616734.2013.830388 24299133

[B34] Meins E. Fernyhough C. Arnott B. Leekam S. R. de Rosnay M. (2013). Mind-mindedness and theory of mind: mediating roles of language and perspectival symbolic play. *Child Dev.* 84 1777–1790. 10.1111/cdev.12061 23432622

[B35] Meins E. Fernyhough C. de Rosnay M. Arnott B. Leekam S. R. Turner M. (2012). Mind-mindedness as a multidimensional construct: appropriate and nonattuned mind-related comments independently predict infant-mother attachment in a socially diverse sample. *Infancy* 17 393–415. 10.1111/j.1532-7078.2011.00087.x 32693485

[B36] Meins E. Fernyhough C. Fradley E. Tuckey M. (2001). Rethinking maternal sensitivity: mothers’ comments on infants’ mental processes predict security of attachment at 12 months. *J. Child Psychol. Psychiatry* 42 637–648. 10.1111/1469-7610.00759 11464968

[B37] Miller S. E. Marcovitch S. (2015). Examining executive function in the second year of life: coherence, stability, and relations to joint attention and language. *Dev. Psychol.* 51 101–114. 10.1037/a0038359 25546598

[B38] Mischel W. Ebbesen E. B. (1970). Attention in delay of gratification. *J. Pers. Soc. Psychol.* 16 329–337. 10.1037/h0029815 5010404

[B39] Mischel W. Shoda Y. Peake P. K. (1988). The nature of adolescent competencies predicted by preschool delay of gratification. *J. Pers. Soc. Psychol.* 54 687–696. 10.1037/0022-3514.54.4.687 3367285

[B40] Moriguchi Y. (2014). The early development of executive function and its relation to social interaction: a brief review. *Front. Psychol.* 5:388. 10.3389/fpsyg.2014.00388 24808885 PMC4010730

[B41] Pederson D. R. Moran G. Sitko C. Campbell K. Ghesquire K. Acton H. (1990). Maternal sensitivity and the security of infant-mother attachment: a q-sort study. *Child Dev.* 61 1974–1983. 10.2307/1130851 2083509

[B42] Rome-Flanders T. Enfant H. M. Cronk C. Gourde C. (1995). Maternal scaffolding in mother-infant games and its relationship to language development: a longitudinal study. *First Lang.* 15 339–355. 10.1177/014272379501504505

[B43] Roskam I. Stievenart M. Meunier J. Noël M. (2014). The development of children’s inhibition: does parenting matter? *J. Exp. Child Psychol*. 122 166–182. 10.1016/j.jecp.2014.01.003 24607865

[B44] Sethi A. Mischel W. Aber J. L. Shoda Y. Rodriguez M. L. (2000). The role of strategic attention deployment in development of self-regulation: predicting preschoolers’ delay of gratification from mother-toddler interactions. *Dev. Psychol.* 36 767–777. 10.1037/0012-1649.36.6.767 11081700

[B45] Soderstrom M. (2007). Beyond babytalk: re-evaluating the nature and content of speech input to preverbal infants. *Dev. Rev.* 27 501–532. 10.1016/j.dr.2007.06.002

[B46] Sulik M. J. Blair C. Mills-Koonce R. Berry D. Greenberg M. (2015). Early parenting and the development of externalizing behavior problems: longitudinal mediation through children’s executive function. *Child Dev.* 86 1588–1603. 10.1111/cdev.12386 26082032 PMC4567899

[B47] Tomlinson M. Cooper P. Murray L. (2005). The Mother-Infant relationship and infant attachment in a South African Peri-Urban settlement. *Child Dev.* 76 1044–1054. 10.1111/j.1467-8624.2005.00896.x 16150001

[B48] Willoughby M. T. Blair C. B. Wirth R. J. Greenberg M. (2010). The measurement of executive function at age 3 years: psychometric properties and criterion validity of a new battery of tasks. *Psychol. Assess.* 22 306–317. 10.1037/a0018708 20528058

[B49] Willoughby M. T. Wirth R. J. Blair C. B. (2011). Contributions of modern measurement theory to measuring executive function in early childhood: an empirical demonstration. *J. Exp. Child Psychol.* 108 414–435. 10.1016/j.jecp.2010.04.007 20553690 PMC2953563

[B50] Xing S. Zhou Q. Archer M. Yue J. Wang Z. (2016). Infant temperamental reactivity, maternal and grandparental sensitivity: differential susceptibility for behavior problems in China. *Early Hum. Dev.* 101 99–105. 10.1016/j.earlhumdev.2016.08.014 27614331

